# Supplementary material for: Modeling the formation of social conventions from embodied real-time interactions
Source: PLoS One. 2020 Jun 22;15(6):e0234434. doi: 10.1371/journal.pone.0234434 (PMC7307740; doi:10.1371/journal.pone.0234434)
Supplement: Attachment — Submitted filename: Response to Reviewers.pdf [file pone.0234434.s003.pdf]

Dear editor and reviewers,

First of all, thank you for your time and efforts to review this manuscript. We truly appreciate the feedback which indeed helped us to improve the quality of the paper. As required, the text of the manuscript has been amended to address the all the points raised (see separate submission with changes highlighted). This document is divided in two sections. In the first one we provide a global response to the main concerns identified by the academic editor. In the second one we provide a detailed answer to each of the reviewer's comments. We hope that the revision is satisfactory and the paper would now be found suitable for publication. Please do not hesitate to contact us with any further questions or queries.

In the reply below, we adopt the following formatting conventions:

- **Reviewer's comments are in bold,**
- Author's replies are in normal text
- ***Extracts from the revised version are in italics, indented right.***

Main concerns identified by the Academic Editor

The main editor has identified two main concerns from the reviews.

1. Please account for the reviewer 2's comments on statistical analysis and comparisons of the human/non-human conditions - mainly the reviewer's comment:

"This requires comparing *model* behavior across the 2x2 conditions (if I recall, this was done correctly in the previous submission I reviewed, and I wasn't sure why it was changed here.) Given this first-order analysis, which is currently missing, the model-human comparisons currently reported could serve as an illuminating follow-up analysis exposing systematic points of deviation from the human data (i.e. motivating discussion of why the model has such poorer stability overall, and poorer efficiency in the *ballistic* condition, where humans still form conventions quite readily?)"

Response:
The comparisons between model conditions have been added as the main results, as suggested by reviewer 2. By applying those changes, the structure of the Results section has been updated (see page 8, par. 5, page 9; page 10, par. 1).

<u>The beginning of the Results section has been modified into:</u>

We report the main results of our model simulations across the 2x2 conditions, which are analyzed using: efficiency, fairness, and stability [43]. For each of these measures, we first present the results of the model and plot them (see Figure 4, bottom panel) in contrast with human data from [23]. Then we perform a detailed pairwise human-model comparison between conditions, we interpret those results, and finally, we analyze the role of each layer of the CRL architecture.

Regarding the efficiency scores, the results of the CRL model followed a normal distribution in the four conditions, so first, a one-way ANOVA was performed, showing a statistically significant difference between groups (F (3, 46) = 755, $p < .001$). Post-hoc independent samples t-tests showed that dynamic (high and low) conditions ($M = 0.88$, $M = 0.86$) are as well significantly more efficient than their ballistic counterparts ($M = 0.46$, $M = 0.45$); between both high (t (98) = -33, $p < .001$) and low conditions (t (98) = -33, $p < .001$). We observe the same statistical tendencies than in the human benchmark data (see Figure \ref{model_results}, top panel).

As for the fairness scores of the model, a one-way ANOVA showed a statistically significant difference between groups (F (3, 46) = 6.88, $p < .001$). Post-hoc independent samples t-tests showed that, as in the benchmark data, the high ballistic condition ($M = 0.50$) is significantly less fair than the two dynamic conditions, high (t (98) = -3.73, $p < .001$) and low (t (98) = -3.99, $p < .001$), both with a mean fairness score of 0.68. On the other hand, there is no statistically significant difference between the low ballistic condition ($M = 0.61$) and the low dynamic ($p = .086$), but, as opposed to the human results, the low and high ballistic conditions of the model are significantly different (t (98) = -2.17, $p < .05$). Again, the same statistical tendencies are observed in human

and model data (see Figure \ref{model_results}, middle panel).

As for stability, since the results of the four conditions showed a non-Gaussian distribution, a non-parametric Kruskal-Wallis H-test was performed, showing a statistically significant difference between groups (H (3) = 42.3, p < .001). Post-hoc Mann-Whitney U-tests showed that the difference between ballistic low (M = 1.18) and dynamic low (M = 1.17) conditions is not statistically significant (p = .10), but that the dynamic high condition (M = 1.09) was significantly more stable than the ballistic high (M = 1.16; p < .001). There was as well a statistically significant difference between the two dynamic conditions (p < .001), but not between the two ballistic (p = .26). Similar statistical tendencies are observed in humans and model (see Figure \ref{model_results}, bottom panel).

Now we perform a pairwise statistical comparison between human and model results to analyse the main points of deviation from the human data.

2. Also, account for the specific comments of Review on how to reorganize the material for better understanding. Reviewer 1 has here useful suggestions on clarifying the motivation of your paper for general audience, for example by adding examples, and including relevant literature on social norms. Please follow these suggestions as well. Also, correct non-working gitlab link(s).

Response:

All the points emphasized by the Associate Editor regarding Reviewer 1 comments have been addressed. Moreover, all the references provided by Reviewer 1 have been incorporated. Please find a point by point reply below.

Replies to the reviewer’s comments

Below we provide detailed to answer to all the reviewer’s comments.

Reviewer 1

The paper studies the role real time control and learning play in the formation of social conventions by using different computational models (simulations) and compare the outcome of these simulations with data from human interactions.
The authors show that in order to reach stable – across round - coordination an adaptive layer in the algorithm is required.
The paper is well written, methodologically sound and makes an interesting contribution. I summarize minor comments (in no particular order) below:

1. Regarding the motivation and literature on social norms and human behaviour, I believe you are missing several papers (summarized in under literature). These papers could help to better motivate the paper with respect to how human cooperation and social norms evolve over time (see, e.g., Cialdini and Trost (1998), Fehr et al. (2002), Fehr and Fischbacher (2003,2004) – which is, I believe, important to know before studying how computer algorithms may mimic (or even outperform)

human behaviour. This literature may also help to give examples in real life where social norms play an important role (see, e.g., the environmental domain (Allcott (2011) and Brülisauer et al. (2018)).

Response:
Several paragraphs of the introduction have been added to integrate the suggested literature regarding the formation and evolution of social norms (see page 1, par. 1,2).

<u>The updated Introduction has been modified into:</u>

> ***Our life in society is often determined by social conventions that affect our everyday decisions. From the side of the road we drive on to the way in which we greet each other, we rely on social norms and conventions to regulate these interactions in the interest of group coordination. Seemingly stable, those norms are not carved in stone, but can evolve and change over time, as seen, for instance, in energy consumption patterns of a population [1, 2]. Moreover, small variations in individual behavior driven by social norms can have huge impact on the environment when implemented at a global scale. Among most recent examples of rapidly emerging social norms with unprecedented consequences one might refer to the social distancing convention adopted as one of the major ways to fight the spread of COVID-19.***
>
> ***But what is a convention, how is it formed and maintained over time? Although some forms of cooperative behaviors are observed across different animal and insect species, current evolutionary models alone do not seem to provide sufficient explanation of why humans but not other animals exhibit large-scale cooperation among genetically unrelated individuals [3]. Behavioral evidence suggests that "strong reciprocity" - defined as a tendency to voluntarily cooperate, if treated fairly, and to punish non-cooperators - [4] can account for this uniquely human type of cooperation. Such conditional cooperation allows humans to reach conventions, although various groups can differ greatly in particular social norms adopted. This variability in social conventions also serves another function: promoting conformity within groups and heterogeneity across groups [5].***

2. The motivation of the paper could be more clear. This is a general interest journal and you might have readers who are also from other disciplines and not familiar with the literature. In this regard, I would like to see a better motivation of why your research is important. It is clear that human cooperation is important. It is also obvious that algorithms play an important role – why, however, should we be interested in algorithms that mimic human behaviour? To me, your contribution would be to better understand human corporation per se but it is not so clear in the current version of the manuscript. It is also not clear why we would need an algorithm for this? What can we learn from the algorithm that we cannot learn from experiments with humans (in the lab or in the fmri, for example). It is also not clear why exactly “a model that can account for how lower-level dynamic processes interact with

higher-level (strategic) cognitive processes" is exactly the next step needed. It would be good if the authors could elaborate more on this point. Moreover, I would like to see where such algorithms may be applied in the real world – if there is any application. Furthermore, why would we be interested in algorithms (for application) that are similar to humans? Should we not compose algorithms that outperform humans with regard to fairness and efficiency, too?

Response:
Several paragraphs of the introduction have been added to improve the motivation of the paper (see page 2, par. 6,7; page 3, par. 1).

<u>The updated Introduction has been modified into:</u>

> ***However, despite the substantial amount of behavioral studies and the advances in the development of more ecologically valid setups, a multi-level theoretical account of mechanisms driving coordination is still missing. A substantial step in this direction could be the development of algorithms modelling observed human behaviors. Simulations derived from these algorithms can be used to test predictions and provide insights on the possible mechanisms behind cooperation which cannot be directly validated by behavioral or neurophysiological data. Moreover, these computational models provide us with a fast way of exploring the vast range of possible experimental conditions and as well help us overcome limitations of human studies related to costs of subject participation.***
>
> ***These models can be used at later stages not only for prediction and validation of the existing theories but also can be applied for control of artificial intelligent agents. If we aim to integrate robots or intelligent machines into our daily lives, we have to provide them with cognitive models that are able to learn and adapt to our social norms and practices. In order to do that, the algorithms governing the agent must integrate high-level information such as rules and plans with embodied information relevant for acting in the real world. One fundamental step in this direction will be a model that can account for how lower-level dynamic processes interact with higher-level (strategic) cognitive processes: incorporating real-time components will not only add ecological validity, but also can bootstrap learning through solving the sampling inefficiency problem and reducing the time it takes for the model to achieve acceptable performance levels, often seen as a major drawback of the machine learning methods [24].***

3. I would also like to see a better motivation of the restrictions applied to the different algorithms. The TD, for example, focusses on long term reward. Would incorporating an algorithm that solely focuses on short term reward change results (I know the focus is coordination over time but there is a substantial literature showing that humans often act irrationally and focus on the present rather than thinking long term and considering future interactions/outcomes (e.g., present bias). I am not asking for such simulations – but I would like to have more focussed discussion on the

properties of the algorithms and what might change if they have different properties or, e.g., why considering different properties is of minor importance.

Response:
We provided better motivation for the selection of the TD learning algorithm in the methods section (see page 6, par. 1,2).

The updated Methods section has been modified into:

> ***The agent's Adaptive layer (AL) is based on a model-free reinforcement learning algorithm that endows the agent with learning capacities for maximizing accumulated reward. More specifically, we use an Actor-Critic Temporal Difference Learning algorithm (TD-learning) which is biologically grounded in temporal difference models of animal learning [3][4][5][6][7]. Our implementation is similar to the one described in [46] and is adapted to operate on discrete state and action spaces.***
>
> ***Functionally, it determines the agent's action at the beginning of the round, based on the state of the previous round and its policy. The possible states S are three: high, low and tie; and they indicate the outcome of the previous round for each agent. That is, if an agent got the high reward on the previous round, the state is high; if it got the low reward, the state is low; and if both agents went to the same reward, the state is tie. The actions A are three as well: go to the high, go to the low and none. The parameter values reported below have been obtained through a parameter search aimed at fitting the behavioral data in [23].***

Moreover, we have added a paragraph to the discussion addressing the issue and pointed to related work in which we report a detailed study on model parameters exploration (see page 14, par. 1,6).

The updated Discussion section has been modified into:

> ***Furthermore, there is a biological correspondence of the functions identified by modules of the CRL architecture. Computations described by temporal difference learning are related to the temporal difference model of animal learning [54, 55, 56] and have been found in the human brain, particularly in the ventral striatum and the orbitofrontal cortex [57].***
>
> ***[...]***
>
> ***For future work, there are several directions in which we can continue to develop research presented in this paper. One obvious extension would be to make the CRL model social. This could be done by representing its partner as an intentional agent or trying to predict and learn what its partner is going to do (as inverse RL, or Bayesian convention formation theories do). Such extension would try to account for individual fixed effects observed in this type of game-theoretical scenarios where different levels of recursive reasoning have been reported [64].***

In this paper we have chosen a TD-learning algorithm as it is considered a validated model of animal learning, tuning its parameters through a search that allowed us to get best fit to data. But the CRL architecture allows for the implementation of other algorithms, as the one shown in an extension of this work [65] that integrates loss aversion bias into a Q-learning algorithm. Therefore, a fruitful avenue for further research would be a more in-depth exploration of the algorithmic and parameter space of the Adaptive layer.

4. Since I am not from the field, I do not want to/and cannot judge the implication of the models itself. What I am missing though is a clear explanation why the TD is only applied in the ballistic setting. Additionally, would it not make sense to compare the models directly, too.

Response:
This might have been a confusion: TD-learning is a component of the Adaptive layer, so it is operating in both conditions (ballistic and dynamic). We were naming the model-ballistic condition 'TD' since the model only operates with the Adaptive layer in this condition. Since it has created confusion, we have changed the labels so that the distinction is clearer now. Moreover, we have added a whole section to the results, directly comparing the models as requested (see abovementioned response to Academic Editor's main points and Figure 5).

5. Statistical testing: you use non-parametric test throughout the paper (and present no test in Section 3.1. but refer to statistical significance of results. I would appreciate if you could show the robustness of the results also in parametric regressions (can you account for individual fixed effects when using the human data?). Moreover, please also provide results for the test performed in Section 3.1

Response:
Non-parametric tests were only used where the normality distribution assumption was violated. Parametric tests were performed in the rest of the cases. As for the results of the tests performed in Section 3.1, they have been added to the manuscript in page 11, par. 3,4; page 12, par. 1).

<u>The updated Results - Model Comparison section has been modified into:</u>

In terms of efficiency, a one-way ANOVA showed a statistically significant difference between models in both high (F(2, 47) = 849.47, p < .001) and low conditions (F(2, 47) = 659.98, p < .001). The post-hoc analysis shows that both the reactive-only (M = 0.46, p < .001) and adaptive-only models (M = 0.46, p < .001) obtained a significantly lower score in this metric compared to the whole CRL architecture (M = 0.88). This is also true for the low condition (reactive M = 0.51, p < .001; adaptive M = 0.45, p < .001; CRL M = 0.86). So overall, agents exclusively dependent on one layer perform worse, as we can see in Figure 7 (left panels). This drop in efficiency is caused by a higher amount of rounds that end up in ties, in which both agents do not receive any reward. On the Fairness metric, the one-way ANOVA also showed statistically significant differences in the two conditions; high (F(2, 47) = 13.07, p < .001) and low (F(2,

47) = 5.25, p < .01). In this case, however, the main differences are found among the ablated models, as shown by the post-hoc analysis of both high (reactive M = 0.71; adaptive M = 0.50, p < .001) and low conditions (reactive M = 0.74; adaptive M = 0.61, p < .001).

The results in the Fairness score of the ablated models are comparable to the ones of the complete CRL model. However, note that these results are computed from fewer rounds, precisely due to the high amount of ties reached by the ablated models (fairness computes how evenly the high reward is distributed among agents). Finally, regarding Stability, the normality tests showed non-Gaussian distribution, therefore Kruskal-Wallis nonparametric test was performed, showing significant differences among the three models in the high ((H(2) = 36.82, p < .001) and low ((H(2) = 51.03, p < .001)) conditions. With the post-hoc Mann-Whitney U-tests, we observe that both ablated models are significantly less stable than the CRL model (M = 1.09) in the high payoff condition (reactive M = 1.17, p < .001; adaptive M = 1.16, p < .001).

As for the low condition, the results indicate that reactive model (M = 1.25) performs significantly worse in terms of stability when compared to the adaptive (M = 1.18, p < .001) and the CRL models (M = 1.17, p < .001). These results show that overall the ablated models are less stable than the CRL model, as indicated by their higher values in surprise (see Figure 7, right panels). From these model ablation studies we can conclude that any of the layers working alone leads to more unstable and less efficient results.

6. Discussion: The discussion should be a bit more broad. In the paper, the authors concentrate on a very specific game – which is important – but also consider certainty in payoffs. In many situations, however, payoffs are uncertain which has consequences for cooperation (see, e.g., Xiao and Kunreuther, (2016)). It would be desirable if you could at least mention this as a further limitation of your algorithms which work only under certainty of payoffs.

Response:
The uncertainty of payoffs has been added to the discussion as a limitation of the current model (see page 14, par. 3).

<u>The Discussion section has been modified into:</u>

Regarding the limits of the model, we observe that the CRL model still does not reach human level performance in terms of stability. Although the model is quite sample-efficient and reaches good performance in very few trials, it is clear that it does not learn at the same rate as humans. This is because the model-free algorithm implemented at the Adaptive layer obviously does not capture the cognitive complexity of strategic human behavior. For instance, people can inductively abstract to a 'turn-taking' strategy while the adaptive layer would have to separately learn policies for the 'up' and 'down' states from scratch. This can be clearly seen when the model is compared to human performance in the ballistic conditions, where only the Adaptive layer is active. It is also worth mentioning that in the current paper the model has only been

tested in a game where payoffs are fixed, therefore another challenge would be implementing a scenario where payoffs are uncertain. This has been shown to affect cooperation in behavioral experiments [62].

Reviewer 2

The authors present a model of how agents coordinate in real time, which integrates a globally adaptive, TD-learning algorithm with a locally reactive, embodied mechanism. This model is evaluated on continuous-time and discrete-time versions of a benchmark task that has posed a challenge for classical models relying purely on game theoretic machinery. The model is shown to successfully account for patterns of convention formation as a function of the 'stakes' encoded in the game's payoffs by learning to rely more or less on the adaptive layer, and ablation studies show that both 'strategic' and 'embodied' components are needed.

This is highly original modeling work that should be of great interest and impact to multiple research communities, and I highly recommend acceptance. I've been invited to review an earlier iteration of this work, and while I felt quite positive about the paper in that previous cycle, I appreciate that the authors have considerably strengthened the presentation for this submission. The new introduction, in particular, does a great job of cutting to the heart of the contribution, and I greatly appreciate the inclusion of a full set of ablation studies to clarify the contributions of the different architecture components.

In addition to the relatively minor suggestions noted below, I think the manuscript would be improved by one more significant revision: I'm concerned that the results in Section 3 are not reporting the relevant comparisons. Currently, all of the tests are directly pairwise between human and model at each factorial condition, where a significant effect is taken as evidence of a mismatch, and a non-significant effect is taken as evidence that the model is matching human scores. Under the logic of null-hypothesis testing, however, a failure to reject the null hypothesis should not be taken as evidence in favor of the null hypothesis, and any of these numbers could hypothetically be made significant by increasing the number of simulations to a sufficiently high N.

In my view, the first-order question of interest is *not* whether the model gets exactly the same numeric scores as humans for each condition but whether it produces the same qualitative pattern *across* conditions, i.e. whether the model has higher efficiency/fairness in the dynamic condition than the ballistic condition, and whether there is an interaction with payoff for stability. This requires comparing *model* behavior across the 2x2 conditions (if I recall, this was done correctly in the previous submission I reviewed, and I wasn't sure why it was changed here.) Given this first-order analysis, which is currently missing, the model-human comparisons currently reported could serve as an illuminating follow-up analysis exposing

systematic points of deviation from the human data (i.e. motivating discussion of why the model has such poorer stability overall, and poorer efficiency in the *ballistic* condition, where humans still form conventions quite readily?)

Response:
The comparisons between model conditions have been added as the main results, following reviewer 2 suggestion. Following the above-mentioned points, the structure of the Results section has also been updated (see abovementioned response to Academic Editor's main points).

Specific comments:
*** the 'inhibitor' function i connecting the two layers is a key component of the proposed architecture but it is not explicitly described in section 2.2 of the main text (only in the caption of Fig. 2 and informally in the "multi-agent simulations" section). Presumably the mechanism connecting the layers is a general feature of the architecture, not just a minor detail of the simulations -- this is also important for understanding the "agents rely more on the adaptive layer when stakes are high" result. I would like to see the description buried in the "In the dynamic condition..." paragraph of section 2.3 moved to the main architecture presentation in 2.2 (possibly as a new subsection 2.2.3, or just as part of the adaptive layer section).**

Response:
The structure of the section 2.2 has been updated with the inhibitor description as part of the Adaptive layer description (see page 7, last paragraph).

<u>The Methods section has been modified into:</u>

> ***The last component of the Adaptive Layer is an inhibitor function, which serves as a top-down control mechanism interfacing between the adaptive and reactive components of the architecture. This function regulates the activity of the reactive control loops based on the action selected by the TD-learning algorithm. In the case that the action selected is go to the high, the inhibitor function will shut down the low reward seeking behavior, allowing the agent to focus only on the high reward. Conversely, if action were go to the low, the reactive attraction to the high reward will be inhibited. Finally, in the case of the none action, all reactive behaviors will remain active. For a video showing the Adaptive layer in action, see https://youtu.be/aAvJ3ukvaac***

*** Again, as an organizational idea, I might move section 2.3 (with the details of the simulation setups) to the beginning of section 3, to make a cleaner distinction between the general CRL formulation and the specifics of the simulations. When reading the results, I was finding myself confused about, for example, how many simulated games the model statistics were based on.**

Response:
Section 2.3 has been moved to the beginning of section 3 as suggested (see page 8, par. 1,2,3).

<u>The Results section has been modified into:</u>

> ***3. Results***
> ***Multi-Agent Simulations***
> ***We follow, as in the Battle of the Exes benchmark [23], a 2x2 between-subjects experimental design. One dimension represents the ballistic and dynamic versions of the game, whereas the other dimension is composed of the high and low difference between payoffs. Each condition is played by 50 agents who are paired in dyads and play together 50 rounds of the game if they are in one of the high payoff conditions (ballistic or dynamic), or 60 rounds if they are in one of the low payoff conditions. Regarding the task, we have developed the two versions (ballistic and dynamic) of the Battle of the Exes in a 2D simulated robotic environment (see Figure 3B for a visual representation). The source code to replicate this experiment is available online at: https://github.com/IsmaelTito/CRL-Exes. In the ballistic condition, there is no possibility of changing the action chosen at the beginning of the round. Therefore, agents only use the Adaptive layer to operate. The two first actions (high and low) will take the agent directly to the respective reward spots, while the none action will choose randomly between them. In each round, the action at chosen by the AL is sampled according to P(A = at|S = st), where st is the actual state observed by the agent. In the dynamic condition, however, agents can change their course of action at any point during the round. Here, the agents use the whole architecture, with the Adaptive and the Reactive layer working together (see Figure 2). The rules of the game are as follows: A round of the game finishes when one of the agents reaches a reward spot. If both agents are within the white circle area when this happens, the result is considered a tie, and both get 0 points. The small spot always gives a reward of 1, whereas the big spot gives 2 or 4 depending on the payoff condition (low or high respectively, see Figure 1). The reward spots are allocated randomly between the two positions at the beginning of each round.***

*** It would be helpful to include some supplementary videos of a few games played by agents, to give an interested reader a more qualitative sense of their behavior in various conditions (e.g. how collision avoidance and reward seeking interact when purely using the reactive-layer, how the adaptive-layer modifies this behavior, etc.)**

Response:
The videos showing the reactive layer operating alone and the whole model (adaptive+reactive) have been uploaded. The links to the video files can be found in the text in page 5, last paragraph, and page 7, last paragraph).

<u>Links to the videos:</u>

https://www.youtube.com/watch?v=QRx1ImkL9P4
https://www.youtube.com/watch?v=aAvJ3ukvaac

*** Fig. 6 would be easier to read if (a) the facets corresponded to Fig. 4, where the low- and high- were split out into the top and bottom rows, (b) each facet had exactly three bars, showing CRL, Adaptive-only, and Reactive-only, to quickly read off the 'contribution' of each (currently the reader has to go back and forth between the y-axis of the top and bottom row to judge which of the lesions had more of an impact on efficiency.**

Response:
One of the optional modifications of the figure has been implemented (option b). Now each facet has three bars. Please also note that Fig. 6 has now become Fig. 7 after the addition of previous changes in the results section.

*** I was interested in how the 'reliance on adaptive layer' shown in Fig. 7 is learned over the course of a game; presumably the model gradually learns on early rounds that a 'none' action leads to a higher payoff on average? Is there any evidence of path-dependence, where the reactive-layer 'bootstraps' conventions, avoiding long runs of ties in early rounds? Perhaps a couple sentences could be added to clarify the mechanism giving rise to the reliance?**

Response:
We have added some clarifications regarding the mechanisms behind the reliance on the Adaptive layer (see page 13, par. 1).

<u>The Results section has been modified into:</u>

> ***Considering that there are only 3 possible actions ('go high', 'go low', 'none'), if the Adaptive layer is randomly choosing the actions, we should observe that the agent selects each action, on average, the same amount of times. That means that prior to any learning, at the beginning of each dyad, the reliance on the Reactive layer would be 33% and the reliance on the Adaptive layer 66%. Starting from this point, if our hypothesis is correct, we will expect to observe an increase in the reliance on the Adaptive layer as the payoff difference increases. As expected, the results confirm, as seen in Figure 8, that there is a steady increase in the percentage of selection of the Adaptive layer as the payoff difference augments. Such increasing reliance on the Adaptive layer is possible due to Reactive layer actively avoiding ties in the early trials, thus facilitating the acquisition of efficient policies.***

*** The gitlab link in the paper seems to be broken (gitlab.com/specslab gave a 404 error)**
Link corrected. The new link is: https://github.com/IsmaelTito/CRL-Exes

*** Typo: "Although there has been progress insofar many …" (should be “insofar as many”)**
Done.

*** Typo: "avoid colliding between each other" (should be either “avoid collisions between” or “avoid colliding into each other”)**
Done.
